# Sex differences and associated factors of dietary diversity among older adults in Bangladesh: findings from a nationally representative cross-sectional study

**DOI:** 10.1136/bmjopen-2025-106903

**Published:** 2026-02-04

**Authors:** Fahmida Akter, Md Mokbul Hossain, Abu Ahmed Shamim, Tanmoy Sarker, Mehedi Hasan, Abu Abdullah Mohammad Hanif, Mohammad Aman Ullah, Malay Kanti Mridha

**Affiliations:** 1 Centre for Non-communicable Diseases and Nutrition, BRAC James P Grant School of Public Health, BRAC University, Dhaka, Bangladesh; 2 National Nutrition Services, Institute of Public Health Nutrition (IPHN), Dhaka, Bangladesh

**Keywords:** Aging, NUTRITION & DIETETICS, Cross-Sectional Studies

## Abstract

**Abstract:**

**Objectives:**

To assess the sex-specific status of dietary diversity (DD) and its associated factors among older men and women (aged ≥60 years) in Bangladesh.

**Design:**

A nationally representative cross-sectional survey was conducted in 2018–2019 on food security, nutrition and health of older adults of both sexes.

**Setting:**

Data were collected from 82 clusters (rural 57, non-slum urban 15 and slum 10) from all eight administrative divisions of Bangladesh.

**Participants:**

A total of 4817 participants aged ≥60 years (2482 men and 2335 women) were enrolled.

**Measures:**

A list-based (24-item) recall method was employed, and the items were later aggregated into 10 food groups. The outcome variable was the prevalence of inadequate DD, defined as consumption of less than 5 food groups out of 10.

**Results:**

Men and women had mean DD scores of 4.33±1.6 and 4.13±1.7, respectively. Women had a higher prevalence of inadequate DD than men (61.2% vs 56.3%, p=0.028). Among the food groups, women consumed significantly less fish and eggs than men (fish: 50.96% vs 57.76%, p=0.002; eggs: 17.13% vs 22.10%, p=0.004). Poor education, rural dwellings, living in homes with fewer individuals and living in food-insecure households were associated with inadequate DD in both sexes. Furthermore, among men, older age and not being currently married, and among women, lower age and living in households with middle wealth status were associated with inadequate DD.

**Conclusions:**

The study found that older women consume relatively less diverse diets than men, and several sociodemographic factors influence inadequate DD among older adults of both sexes in Bangladesh. These factors should be considered while developing policies and programmes to promote equitable access to a diversified diet and improve geriatric health and nutritional outcomes.

**STRENGTHS AND LIMITATIONS OF THIS STUDY**

- This study used a nationally representative sample of older adults from rural, non-slum urban and slum areas across Bangladesh, enhancing the generalisability of the findings to the broader older adult population.
- Dietary information was self-reported, which may have introduced a social desirability bias.
- The study did not account for several factors that have been shown to affect the nutritional status of older adults, including oral health, chronic diseases such as diabetes and hypertension, cognitive status and social support.

## Introduction

 The United Nations declared 2021–2030 as the Decade of Healthy Ageing^1^ to prioritise the health and well-being of older adults. Numerous studies have shown the importance of diet quality and diversity in healthy ageing and delaying the onset of age-related health problems.^2 3^ Dietary diversity (DD), one of the indicators for assessing a person’s overall diet, is widely acknowledged as a proxy measure of diet quality and is defined as the range of food groups consumed during a specific reference period.^4^

Age-related health conditions, such as malnutrition, cognitive dysfunction and metabolic syndrome, are associated with DD.^5^,^7^ The prevention of non-communicable disease-related disabilities and complications, improvement of living standards and promotion of healthy ageing have all been linked to improved dietary patterns and proper nutrition.^8 9^ A poor diet is a modifiable risk factor for osteoporosis, which is a leading cause of fractures and long-term disability in the elderly.^10^ Moreover, numerous studies have demonstrated that higher DD is associated with a reduced risk of mortality.^11 12^

Globally, many studies have been conducted on diet quality and DD in children, adolescents and women; however, very little has been done on older adults. A study conducted in Botswana among those aged 60 years or older suggested a low variety of foods consumed by older adults.^13^ Older adults in Thailand with better financial standing, advanced education and city residency consume a wider variety of foods, whereas those living alone consume less diverse foods.^14^ A study from India revealed inadequate DD among individuals ≥60 years of age and reported that place of residence, family structure, age and profession were associated with DD.^15^

Although the proportion of people aged ≥60 years is predicted to reach 21.3% by 2050, with the rising trend of life expectancy in Bangladesh,^16^ there has been limited research on DD and its predictors in this age group. Moreover, sex differences in dietary consumption and sex-specific determinants of DD remain to be explored. This lack of evidence severely restricts evidence-based policy formulation and intervention in older adults. Therefore, we aimed to assess the nationwide prevalence of inadequate DD and its determinants among men and women aged ≥60 years in Bangladesh.

## Methods

### Study design, sampling and study participants

We used data from a nationally representative cross-sectional survey conducted in 2018–2019 on food security, nutrition and health of six population groups, including older adults (aged ≥60 years) of both sexes. We collected data from 82 clusters (rural 57, non-slum urban 15 and slum 10), randomly selected from all eight administrative divisions of Bangladesh. This analysis included 4817 older adults (2482 men and 2335 women) who were enrolled in this survey, interviewed and assessed anthropometrically. Details of the study design, sampling methodology, sample size determination and data collection procedures have been described elsewhere in multiple previous papers.^17^,^19^ We reported this study in accordance with the Strengthening the Reporting of Observational Studies in Epidemiology guidelines (online supplemental file 1).

### Study procedures

We collected data through face-to-face interviews using a structured questionnaire and performed anthropometric measurements after obtaining written informed consent from the participants. The data collectors captured the data directly into a template developed using SurveyCTO, a digital data collection application, with the help of tablet computers. Detailed procedures regarding questionnaire finalisation, anthropometric data collection and quality control have been previously outlined.^17^,^19^

### Outcome variable

For dietary data collection, we employed a list-based (24 food items) recall method for the last 24 hours and aggregated these items into 10 food groups (grains, white roots, tubers and plantains; pulses: beans, peas and lentils; nuts and seeds; dairy; meat, poultry and fish; eggs; dark green leafy vegetables; other vitamin A-rich fruits and vegetables; other vegetables; and other fruits), following the Food and Agriculture Organization (FAO) guidelines for estimation of DD of women.^20^ Diet quality and diversity were estimated by the consumption of foods from various food groups, the prevalence of inadequate DD (defined as consumption of less than 5 food groups out of 10) and the DD score (consumption of the actual number of food groups) using a 24-hour recall period according to the FAO guidelines.^20^ The cut-off used to define inadequate DD has not been specifically validated for older adult populations, although it has been used for other age and sex groups.^21 22^

### Explanatory variables

We included several individual (sociodemographic, lifestyle and nutrition) and household-level variables as covariates. The explanatory variable includes age in years (60–64, 65–69 and ≥70); sex (man/woman); educational attainment (no education: grade 0, primary incomplete: grades 1–4; primary complete and above: grade 5 and above); marital status (currently married and others includes single, separated and widow); monthly average individual income (no income, below median: <8000 Bangladeshi taka (BDT)/month, median or above: ≥8000 BDT/month); sedentary time per day (0–240 min/day, 241–360 min/day, >360 min/day); current daily user of any combustible tobacco (yes/no) products; current daily user of any smokeless tobacco (yes/no); body mass index (underweight: <18.50 kg/m^2^, normal: ≥18.50 to <23 kg/m^2^, overweight and/or obese: ≥23 kg/m^2^);^23^ place of residence (rural, non-slum urban, slum); religion (Islam and other than Islam); household size (1–3 members, 4–5 members, ≥6 members); household headship (female headed/male headed); household food security status (food secure, mild food insecure, moderate food insecure, severe food insecure);^24^ and household wealth in quintile (poorest, poorer, middle, richer and richest).

Food security was assessed using the Household Food Insecurity Access Scale (HFIAS), which measures three domains of food insecurity: (1) anxiety and uncertainty about food supply, (2) insufficient food quality and (3) insufficient food intake.^24^ Following the HFIAS guidelines, nine occurrence questions with a 4-week recall period were asked, with responses coded from zero to three (never to often). Using the HFIAS prevalence indicator, households were classified as food secure, and mildly, moderately and severely food insecure. Wealth was assessed using standard MEASURE-DHS questions.^25^ Household assets and characteristics, including amenities, cooking fuel, water source, toilet type and housing materials, were analysed using principal component analysis to construct a composite wealth index following DHS methods. The final index was divided into five quantiles.

### Statistical analysis

Stata V. 17.0 (StataCorp) was used for data management and analyses. First, we performed a descriptive analysis to report the background information of the respondents and their consumption of foods from different food groups. We then estimated the weighted prevalence of inadequate DD. For weighting, the inverse probability weight was used at every sampling stage. We performed Pearson’s χ^2^ test to examine whether the indicators varied significantly between men and women. Subsequently, we performed bivariate logistic regression to assess the relationship between inadequate DD and each explanatory variable among men and women. Finally, we performed multivariable logistic regression after considering collinearity and excluding explanatory variables with a p value >0.2 in the bivariate analysis.^26^ We assessed multicollinearity among the explanatory variables using a correlation matrix before performing multivariable logistic regression. As no strong correlations were identified, no explanatory variables were excluded based on multicollinearity. Therefore, we estimated both crude and adjusted OR and 95% CIs of OR, and a p value of <0.05 was considered statistically significant.

## Results

Approximately one-third of the participants were aged ≥70 years (table 1). Only 25.8% had completed at least primary education, with a notably lower proportion among women (men: 37.3%, women: 13.7%). Overall, 59.4% of older adults were married, and the proportion was significantly lower among women than among men (men: 91.7% vs women: 25.0%). Approximately 37% of men and 3% of women were current smokers. The percentage of daily smokeless tobacco users was 41.4% for men and 57.9% for women. 28% of men and 34.4% of women were either overweight or obese. Notably, 10.7% of men lived in households headed by women, which was significantly lower than the 44.4% of women living in similar situations.

**Table 1:** Distribution of older adults by background characteristics in Bangladesh, 2018–2019

Characteristics	All older adults (n=4817)		Men (n=2482)		Women (n=2335)	
	n	%	n	%	n	%
Age (years)						
60 to 64	2081	43.2	1101	44.4	980	42.0
65 to 69	1144	23.7	624	25.1	520	22.3
≥70	1592	33.0	757	30.5	835	35.8
Educational attainment						
No education (grade 0)	3014	62.6	1214	48.9	1800	77.1
Primary incomplete (grades 1–4)	558	11.6	342	13.8	216	9.3
Primary complete and above (grade 5 and above)	1245	25.8	926	37.3	319	13.7
Marital status						
Currently married	2861	59.4	2277	91.7	584	25.0
Others*	1956	40.6	205	8.3	1751	75.0
Monthly average individual income						
No income	1520	31.6	366	14.7	1154	49.4
<8000 BDT	1820	37.8	988	39.8	832	35.6
≥8000 or more BDT	1477	30.7	1128	45.4	349	14.9
Sedentary time						
0 to 240 min/day	1377	30.8	771	33.4	606	27.9
241 to 360 min/day	1445	32.3	702	30.5	743	34.2
>360 min/day	1654	37.0	832	36.1	822	37.9
Current daily user of any smoking tobacco						
No	3828	79.5	1564	63.0	2264	97.0
Yes	989	20.5	918	37.0	71	3.0
Current daily user of any smokeless tobacco						
No	2438	50.6	1455	58.6	983	42.1
Yes	2379	49.4	1027	41.4	1352	57.9
Nutritional status (BMI)						
Underweight	1100	23.9	589	24.4	511	23.2
Normal	2077	45.1	1145	47.5	932	42.4
Overweight and/or obese	1431	31.1	675	28.0	756	34.4
Place of residence						
Rural	3463	71.9	1835	73.9	1628	69.7
Non-slum urban	807	16.8	394	15.9	413	17.7
Slum	547	11.4	253	10.2	294	12.6
Religion						
Islam	4075	84.6	2113	85.1	1962	84.0
Other than Islam	742	15.4	369	14.9	373	16.0
Household size						
1 to 3 members	1748	36.3	911	36.7	837	35.8
4 to 5 members	1812	37.6	876	35.3	936	40.1
≥6 members	1257	26.1	695	28.0	562	24.1
Household headship						
Female headed	1302	27.0	265	10.7	1037	44.4
Male headed	3515	73.0	2217	89.3	1298	55.6
Household food insecurity status						
Food secure	2798	58.1	1459	58.8	1339	57.3
Mild food insecure	1078	22.4	594	23.9	484	20.7
Moderate food insecure	289	6.0	137	5.5	152	6.5
Severe food insecure	652	13.5	292	11.8	360	15.4

* Others include single, separated and widowed.

BDT, Bangladeshi taka; BMI, body mass index.

Table 2 presents data on DD and diet quality among older adults. Only 41.4% of older adults consumed foods from five or more food groups. The proportion of adequate DD was significantly higher in men (43.8%) than in women (38.8%) (p=0.028). On average, both sexes had relatively similar mean DD score, with men scoring higher (4.33) than women (4.13) (p<0.001). Furthermore, the proportion of men consuming animal-source foods (meat, fish, eggs and dairy) was slightly higher than that of women, showing statistically significant differences in fish (men: 57.76%; women: 50.96%; p=0.002) and egg (men: 22.10% and women: 17.13%; p=0.004) consumption. The differences in the consumption of other nutrient-rich foods (pulses, nuts, seeds, fruits and vegetables) between men and women were not statistically significant.

**Table 2:** Percent distribution of older adults by different indicators of diet quality and diversity in last 24 hours in Bangladesh, 2018–2019

Indicators	All older adults (n=4817) %	Men (n=2482) %	Women (n=2335) %	P value (men vs women)
Consumed foods from ≥5 food groups	41.40	43.75	38.82	0.028
Percent consuming nutrient-rich foods				
(a) Animal-source foods				
Meat, poultry	25.83	27.05	24.47	0.062
Fish (any)	54.52	57.76	50.96	0.002
Egg	19.74	22.10	17.13	0.004
Dairy	29.04	30.41	27.52	0.129
(b) Pulses, nuts and seeds				
Pulses	37.55	39.24	35.69	0.084
Nuts and seeds	4.50	4.45	4.55	0.927
(c) Fruits and vegetables				
Dark green leafy vegetables	34.65	34.27	35.08	0.711
Other vitamin A-rich fruits or vegetables	24.41	24.31	24.52	0.904
Other vegetables	74.70	75.18	74.18	0.541
Other fruits	31.38	32.35	30.31	0.289

	All older adults (n=4817) Mean±SD	Men (n=2482) Mean±SD	Women (n=2335) Mean±SD	
Mean±SD dietary diversity score (DDS) (out of 10 groups)	4.24±1.62	4.33±1.56	4.13±1.67	<0.001
Mean±SD number of fruit/vegetable groups (out of 4 groups)*	1.65±1.01	1.66±0.98	1.64±1.03	0.299

* 4 groups include (1) dark green leafy vegetables, (2) other vitamin A-rich fruits or vegetables, (3) other vegetables and (4) other fruits.

**Figure 1:**
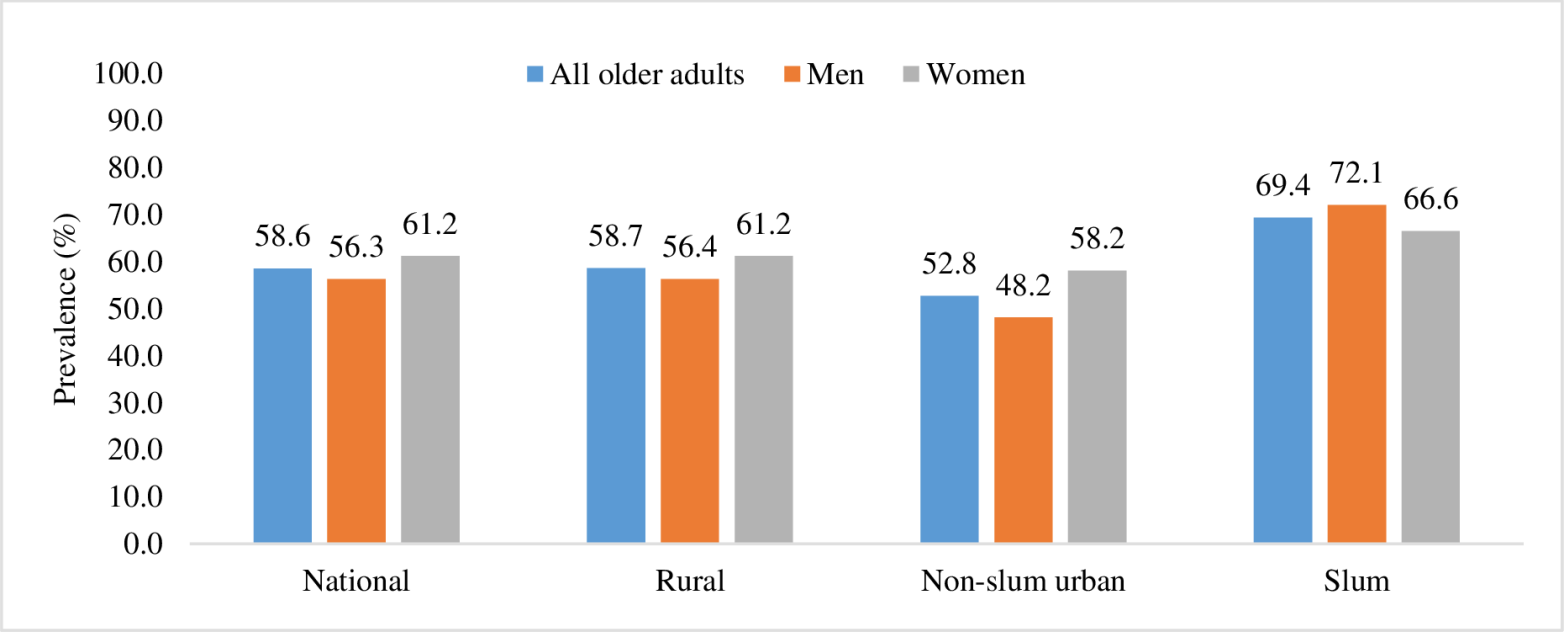
The prevalence of inadequate DD among older adults (who consumed foods from <5 food groups in the last 24 hours) by place of residence in Bangladesh, 2018–2019. DD, dietary diversity.

Figure 1 shows the weighted prevalence of inadequate DD among older adults, offering insights into gender-specific differences and variations across residential settings (rural, non-slum urban and slums). The overall prevalence of inadequate DD among the older adults was 58.6%. Dissimilarities were observed across different residential areas. Overall, the prevalence of inadequate DD was highest among older adults residing in slum areas (69.4%), followed by those living in rural settings (58.7%) and non-slum urban areas (52.8%). In rural and non-slum urban areas, the prevalence of inadequate DD significantly differed between sexes (higher prevalence among women than among men). In contrast, in slum areas, the prevalence was higher among men (72.1%) than among women (66.6%), although the difference was not statistically significant.

Among older men, the multivariable logistic regression analyses identified that being within the 65–69 years age group (AOR 1.38, 95% CI 1.12 to 1.70); poor formal education (AOR 1.61, 95% CI 1.32 to 1.97 for no education; AOR 1.66, 95% CI 1.27 to 2.18 for incomplete primary education); not currently being married (AOR 1.46, 95% CI 1.05 to 2.02); and residing in food-insecure households (AOR 1.67, 95% CI 1.34 to 2.07 for mild food insecurity; AOR 2.83, 95% CI 1.85 to 4.33 for moderate food insecurity; and AOR 1.75, 95% CI 1.32 to 2.33 for severe food insecurity) were significantly associated with a higher prevalence of inadequate DD (table 3). On the other hand, we also found a protective effect of residing in non-slum urban areas (AOR 0.63, 95% CI 0.49 to 0.82) and living in larger households (AOR 0.77, 95% CI 0.63 to 0.94 for 4–5 membered household; AOR 0.70, 95% CI 0.56 to 0.87 for households with six or more members) for inadequate DD among older men.

**Table 3:** Bivariate and multivariable analyses of background characteristics with inadequate DD among older men (n=2482) in Bangladesh, 2018–2019

Characteristics	COR			AOR		
	COR	95% CI	P value	AOR	95% CI	P value
Age (years)						
60 to 64	Ref			Ref		
65 to 69	1.30	1.06 to 1.58	0.010	1.38	1.12 to 1.70	0.002
≥70	1.32	1.10 to 1.59	0.003	1.18	0.96 to 1.45	0.122
Educational attainment						
No education (grade 0)	2.09	1.75 to 2.48	<0.001	1.61	1.32 to 1.97	<0.001
Primary incomplete (grades 1–4)	1.98	1.53 to 2.55	<0.001	1.66	1.27 to 2.18	<0.001
Primary complete and above (grade 5 and above)	Ref			Ref		
Marital status						
Currently married	Ref			Ref		
Others*	1.38	1.03 to 1.86	0.032	1.46	1.05 to 2.02	0.024
Monthly average individual income						
No income	1.18	0.93 to 1.50	0.170	0.95	0.73 to 1.24	0.721
<8000 BDT	1.52	1.28 to 1.81	<0.001	1.05	0.86 to 1.27	0.653
≥8000 or more BDT	Ref			Ref		
Sedentary time per day						
0 to 240 min/day	Ref			Ref		
241 to 360 min/day	1.28	1.05 to 1.57	0.013	1.16	0.94 to 1.43	0.163
>360 min/day	1.04	0.86 to 1.25	0.674	0.96	0.79 to 1.18	0.729
Current daily user of any smoking tobacco						
No	Ref			Not applicable		
Yes	1.07	0.90 to 1.26	0.446			
Current daily user of any smokeless tobacco						
No	Ref			Ref		
Yes	1.14	0.97 to 1.34	0.113	1.05	0.88 to 1.25	0.577
Nutritional status (BMI)						
Underweight	1.10	0.89 to 1.34	0.377	0.96	0.78 to 1.19	0.721
Normal	Ref			Ref		
Overweight and/or obese	0.69	0.57 to 0.83	<0.001	0.93	0.76 to 1.15	0.505
Place of residence						
Rural	Ref			Ref		
Non-slum urban	0.46	0.37 to 0.58	<0.001	0.63	0.49 to 0.82	0.001
Slum	1.16	0.89 to 1.53	0.271	1.03	0.77 to 1.38	0.844
Religion						
Islam	Ref			Not applicable		
Other than Islam	1.14	0.91 to 1.43	0.245			
Household size						
1 to 3 members	Ref			Ref		
4 to 5 members	0.76	0.63 to 0.92	0.004	0.77	0.63 to 0.94	0.012
≥6 members	0.66	0.54 to 0.8	<0.001	0.70	0.56 to 0.87	0.001
Household headship						
Female headed	1.26	0.97 to 1.63	0.084	1.27	0.96 to 1.68	0.093
Male headed	Ref			Ref		
Household food insecurity status						
Food secure	Ref			Ref		
Mild food insecure	1.90	1.56 to 2.32	<0.001	1.67	1.34 to 2.07	<0.001
Moderate food insecure	3.25	2.18 to 4.85	<0.001	2.83	1.85 to 4.33	<0.001
Severe food insecure	2.22	1.71 to 2.90	<0.001	1.75	1.32 to 2.33	<0.001
Household wealth						
Poorest	1.55	1.21 to 1.98	0.001	0.83	0.62 to 1.11	0.198
Poorer	1.92	1.49 to 2.48	<0.001	1.10	0.82 to 1.48	0.514
Middle	1.63	1.26 to 2.10	<0.001	0.91	0.68 to 1.22	0.544
Richer	1.12	0.87 to 1.44	0.399	0.82	0.62 to 1.08	0.165
Richest	Ref			Ref		

* Others include single, separated and widow.

AOR, adjusted OR; BDT, Bangladeshi taka; BMI, body mass index; COR, crude OR; DD, dietary diversity.

Multivariable logistic regression also revealed that not having any formal education (AOR 1.60, 95% CI 1.22 to 2.10); residing in food-insecure households (AOR 1.67, 95% CI 1.31 to 2.13 for mild food insecurity; and AOR 2.45, 95% CI 1.59 to 3.78 for moderate food insecurity); and living in middle-wealth quintile households (AOR 1.40, 95% CI 1.04 to 1.88) significantly increased the odds of having inadequate DD among older women in Bangladesh (table 4).

**Table 4:** Bivariate and multivariable analyses of background characteristics with inadequate DD among older women (n=2335) in Bangladesh, 2018–2019

Characteristics	COR			AOR		
	COR	95% CI	P value	AOR	95% CI	P value
Age (years)						
60 to 64	Ref			Ref		
65 to 69	0.77	0.62 to 0.95	0.017	0.76	0.60 to 0.95	0.018
≥70	0.82	0.68 to 0.99	0.041	0.73	0.59 to 0.90	0.004
Educational attainment alternative						
No education (grade 0)	2.01	1.58 to 2.55	<0.001	1.60	1.22 to 2.10	0.001
Primary incomplete (grades 1–4)	1.43	1.01 to 2.03	0.042	1.19	0.82 to 1.74	0.354
Primary complete and above (grade 5 and above)	Ref			Ref		
Marital status						
Currently married	Ref			Not applicable		
Others*	1.02	0.84 to 1.24	0.832			
Monthly average individual income						
No income	0.75	0.58 to 0.96	0.022	0.64	0.49 to 0.84	0.001
<8000 BDT	0.95	0.73 to 1.24	0.725	0.74	0.55 to 0.98	0.036
≥8000 or more BDT	Ref			Ref		
Sedentary time per day						
0 to 240 min/day	Ref			Ref		
241 to 360 min/day	1.26	1.02 to 1.56	0.029	1.13	0.9 to 1.41	0.291
>360 min/day	0.98	0.8 to 1.2	0.869	0.96	0.78 to 1.2	0.748
Current daily user of any smoking tobacco						
No	Ref			Not applicable		
Yes	1.22	0.74 to 2.00	0.444			
Current daily user of any smokeless tobacco						
No	Ref			Ref		
Yes	1.18	0.99 to 1.39]	0.059	1.09	0.9 to 1.3	0.374
Nutritional status (BMI)						
Underweight	1.30	1.03 to 1.63	0.026	1.18	0.93 to 1.5	0.163
Normal	Ref			Ref		
Overweight and/or obese	0.82	0.68 to 1.00	0.052	0.91	0.74 to 1.12	0.373
Place of residence						
Rural	Ref			Ref		
Non-slum urban	0.58	0.47 to 0.72	<0.001	0.81	0.62 to 1.06	0.125
Slum	0.79	0.61 to 1.01	0.062	0.73	0.55 to 0.97	0.029
Religion						
Islam	Ref			Not applicable		
Other than Islam	0.98	0.78 to 1.23	0.846			
Household size						
1 to 3 members	Ref			Ref		
4 to 5 members	0.8	0.66 to 0.97	0.025	0.92	0.74 to 1.14	0.460
≥6 members	0.7	0.56 to 0.87	0.001	0.75	0.58 to 0.96	0.022
Household headship						
Female headed	1.24	1.04 to 1.46	0.014	1.15	0.94 to 1.39	0.173
Male headed	Ref			Ref		
Household food insecurity status						
Food secure	Ref			Ref		
Mild food insecure	1.90	1.52 to 2.38	<0.001	1.67	1.31 to 2.13	<0.001
Moderate food insecure	3.06	2.03 to 4.6	<0.001	2.45	1.59 to 3.78	<0.001
Severe food insecure	1.37	1.08 to 1.74	0.010	1.20	0.91 to 1.57	0.195
Household wealth						
Poorest	1.88	1.43 to 2.46	<0.001	1.28	0.94 to 1.75	0.120
Poorer	2.04	1.56 to 2.66	<0.001	1.36	1.00 to 1.86	0.051
Middle	1.94	1.49 to 2.52	<0.001	1.40	1.04 to 1.88	0.027
Richer	1.28	1.00 to 1.65	0.054	1.14	0.87 to 1.50	0.346
Richest	Ref			Ref		

* Others includes single, separated and widow.

AOR, adjusted OR; BDT, Bangladeshi taka; BMI, body mass index; COR, crude OR; DD, dietary diversity.

Interestingly, the association between age and inadequate DD differed between men and women. Women aged 65–69 years (AOR 0.76, 95% CI 0.60 to 0.95) and those aged 70 years or older (AOR 0.73, 95% CI 0.59 to 0.90) showed lower odds of experiencing inadequate DD, contrary to the findings observed among men. Furthermore, having no individual income or an income less than the median displayed a protective effect against inadequate DD among women. Additionally, women living in slum areas exhibited 45% lower odds of experiencing inadequate DD than those residing in rural areas. Like men, living in households with six or more members reduced the odds of having inadequate DD among women.

## Discussion

The purpose of this study was to estimate the prevalence of inadequate DD and its determinants among men and women aged ≥60 years in Bangladesh. The findings revealed that inadequate DD was highly prevalent among Bangladeshi older adults, with women having a substantially higher prevalence than men. Among the food groups, women consumed significantly less fish and eggs than did men. Moreover, the study revealed sex-specific differences in the strength and direction of the associations between the prevalence of inadequate DD and other explanatory variables. Inadequate DD among older adults was associated with poor education, residing in rural areas, living in households with fewer individuals and living in food-insecure households of both sexes. Specifically, among men, older age and not currently married were associated with inadequate DD. In contrast, among women, younger age and living in households with middle wealth status were significantly associated with inadequate DD.

We reported that Bangladeshi older adults consumed a poorly diversified diet, with older women showing even less DD than older men, highlighting critical public health concerns. These gender disparities in DD, as observed in this study, may reflect broader sociocultural norms and economic inequalities. For example, women often eat last within households, leading to lower dietary intake or limited access to diverse foods, and they may also experience reduced autonomy in food choices. Additionally, older women are more likely to lack financial independence and rely on family members for food, which can limit their access to a variety of foods. In contrast, a study among older adults living alone in Japanese communities found that the prevalence of low dietary variety scores among men was more than twice that among women.^27^ Another study from Brazil reported no statistically significant differences in diet quality between older men and women.^28^ Despite the varying global contexts, our findings underscore the need for targeted interventions that specifically address the unique dietary challenges faced by older adults, particularly in women. Although some sex differences in specific food groups (adequate DD, fish consumption and egg consumption) were statistically significant, most numerical differences across other food groups were small and not clinically meaningful and should therefore be interpreted with caution. Future research should further explore the underlying drivers of this disparity and evaluate strategies to promote equitable food access and improve dietary quality among older adults in Bangladesh.

Our findings revealed that household food insecurity was associated with poor DD among older adults in Bangladesh, which is consistent with observations from studies focusing on older age groups. For instance, an Iranian study among older adults found that participants in food-insecure groups had poor DD compared with the food-secure group.^29^ This association between poor DD and food insecurity has been well documented in other age groups.^21 30^ Food-insecure households tend to allocate a larger share of their limited resources to staple foods (eg, rice) to meet their calorie needs rather than spending on non-staple foods, which is arguably an indicator of DD, resulting in poor DD. However, several studies have found no or an inverse association between poor DD and food insecurity among the elderly.^31^

We found an association between poor educational level and inadequate DD, which is not uncommon. Older adults with no education or those who did not complete primary education had significantly higher levels of inadequate DD than those who had completed primary education. Individuals with higher education levels are more inclined to comprehend the advantages of a diversified diet, have access to diverse sources of nutritional information^32^ and possess greater financial capability or purchasing power than older adults with lower education. A study conducted in Brazil revealed that older individuals with 0–4 years of education consumed a lower range of food groups and had a lower healthy eating index than those with at least 9 years of education.^28^ One potential interpretation is that the lack of or lower educational attainment of individuals may impact their understanding of nutrition, consequently leading to decreased DD.

This study revealed that with an increase in household size, inadequate DD for older individuals decreased. When the number of family members increased, the diversity of diet also increased consistently in both older men and women. Studies from Bangladesh and West Bengal have also found similar evidence, although the population groups were different.^33 34^ Several other studies have also shown that older adults living with more family members, rather than living alone, have significantly higher DD.^14 35^ One possible explanation is that family gatherings during meal time are common in larger households, and older individuals enjoy eating with their family rather than eating alone,^36^ thereby promoting DD. In addition, larger families may have greater food-purchasing power, allowing them to acquire a wider variety of foods. In larger households, there may be more members who take care of older adults.

Our findings highlighted that living in rural areas was a significant factor limiting DD among older adults compared with living in non-slum urban areas for men and slum areas for women. This finding is congruent with several other studies conducted in older age groups.^13 14^ Previously, we also observed that older adults from urban areas consumed more unhealthy foods and sugar-sweetened beverages, indicating the consumption of a variety of healthy and unhealthy foods and beverages among older adults residing in urban areas.^19^ A possible explanation for this is the higher availability and accessibility of diverse foods in urban settings. Compared with rural regions, urban areas have a much larger density of food stores and supermarkets.^37^ In addition, with rapid urbanisation in Bangladesh, people prefer to consume food away from home in restaurants and streets, and non-rice snacks dominate food preferences in urban areas, which may contribute to increased DD.^37 38^ Consequently, older individuals residing in urban settings might benefit from closer proximity to food outlets, thus having greater access to a variety of foods than their rural counterparts.

Our study found that among older men, not being married was linked to higher odds of poor DD. Although the study contexts differed, multiple reasons may account for these findings. One reason could be that men typically depend on their wives for food preparation, and as a result, they might possess comparatively limited knowledge about nutrition and lack meal preparation and cooking skills compared with women.^39 40^ Moreover, different studies have highlighted a positive correlation between marital status and diet quality in older adults.^40^ The dynamics within marital relationships can potentially affect dietary habits through shared meal activities, although this does not guarantee dietary quality. To date, the evidence regarding these factors has not been definitive. There is a lack of studies examining the gender-specific relationship between marital status and diet quality among older adults in Bangladesh, underscoring the urgency for such research initiatives.

Furthermore, the association between DD and household wealth differed between men and women. Among women, a higher likelihood of inadequate DD was observed among those from less-affluent households than among those from the richest households. Notably, this increased likelihood was statistically significant solely among households categorised as middle wealth. However, in men, this association was neither unidirectional nor conclusive. Additionally, we observed that no income or poor individual income reduced the likelihood of inadequate DD among women. However, this association was neither distinct nor statistically significant in men. These findings suggest that financial factors tend to exhibit a more consistent influence on DD among women than men.

In this study, we reported sex-based disparities in the association between DD and age among older men and women in Bangladesh. A cohort study conducted in the UK observed a similar pattern of association between older men and women. Bloom *et al* revealed that while the average diet quality remained stable with advancing age in men, a noticeable decline in overall diet quality was observed in women as they aged.^41^ Further research is needed to find possible explanations in the context of Bangladesh behind the associations identified in our study.

A major strength of this study is the use of a nationally representative sample of older adults drawn from rural, non-slum urban and slum areas across Bangladesh. Data collection was conducted through face-to-face interviews in community settings, rather than in institutional environments such as hospitals or old age homes, thereby enhancing the generalisability of the findings to the wider older adult population. Nonetheless, this study has several limitations. The cross-sectional nature of this study limits the ability to infer causal relationships from the observed associations. Dietary data were self-reported, introducing the possibility of social desirability bias. The study did not account for several factors that affect the nutritional status of older adults, including oral health, chronic diseases such as diabetes and hypertension, cognitive status and social support. In addition, while the study employed a widely used cut-off to define ‘inadequate DD’ (consumption from fewer than 5 of 10 food groups), this threshold has not been validated for older adult populations. Future research is needed to develop and validate an age-appropriate cut-off to more accurately assess DD inadequacy in this demographic group.

## Conclusions

As Bangladesh is undergoing an epidemiological shift characterised by a growing older population, it is imperative to prioritise the overall quality of life of this age group. Improving DD has emerged as a pivotal strategy to promote healthy ageing in older adults. This study contributes to understanding the existing gender disparity in diet quality and identifies the demographic, social and financial determinants of DD among older adults of both sexes in Bangladesh. The results highlighted the significance of living arrangements, such as living in larger households and place of residence; financial circumstances encompassing household food security, wealth status and personal income; and individual characteristics including age, education and marital status among older adults, all crucial factors in ensuring adequate diversified diets. Our results can guide implementers and policymakers to improve the design and implementation of geriatric food and nutrition interventions, policies and strategies to improve dietary practices, particularly focusing on the identified vulnerable socioeconomic groups. These findings can be used as a starting point for future research that estimates the amount of food consumed by older men and women to pinpoint the exact nutrient gap and guide more targeted policies and interventions in this area. Further in-depth research is required for a comprehensive understanding of the factors and mechanisms that influence the dietary practices of older adults in Bangladesh.

## Supplementary material

10.1136/bmjopen-2025-106903 online supplemental file 1

## Data Availability

The data used in this manuscript are available upon request. All such requests can be sent to the Institutional Review Board (IRB), BRAC James P Grant School of Public Health, BRAC University, Dhaka, Bangladesh to the email address: irb-jpgsph@bracu.ac.bd
