# Supplementary material for: Sex differences and associated factors of dietary diversity among older adults in Bangladesh: findings from a nationally representative cross-sectional study
Source: BMJ Open. 2026 Feb 4;16(2):e106903. doi: 10.1136/bmjopen-2025-106903 (PMC12878318; doi:10.1136/bmjopen-2025-106903)
Supplement: Supplementary file 2 [file bmjopen-2025-106903.reviewer_comments.pdf]

PEER REVIEW HISTORY

BMJ Open publishes all reviews undertaken for accepted manuscripts. Reviewers are asked to complete a checklist review form and are provided with free text boxes to elaborate on their assessment. These free text comments are reproduced below.

ARTICLE DETAILS

Title (Provisional)

Sex differences and associated factors of dietary diversity among older adults in Bangladesh: Findings from a nationally representative cross-sectional study

Authors

Akter, Fahmida; Hossain, Md Mokbul; Shamim, Abu Ahmed; Sarker, Tanmoy; Hasan, Mehedi; Hanif, Abu Abdullah Mohammad; Ullah, Mohammad Aman; Mridha, Malay Kanti

---

VERSION 1 - REVIEW

**Reviewer**	**1**
**Name**	**Addae Yaw, Hammond**
**Affiliation**	**Nursing & Midwifery Training Colege, Kpembe, Nursing**
**Date**	**15-Oct-2025**
**COI**	**None**

Title: Sex differences and associated factors of dietary diversity among older adults in Bangladesh: Findings from a nationally representative cross-sectional study

Summary

Thank you for the opportunity to review this manuscript.

This is an important study that was conducted among a population that is often overlooked, yet are becoming a sub-group that requires nutritional attention. The sample size is a strength; however, methodological approaches may limit the thoroughness of this study. Specifically, the non-inclusion of equally important and well-known nutrition-related geriatric factors, such as cognitive status, or the presence or otherwise of chronic diseases (e.g. hypertension, diabetes etc), is a main flaw.

The authors may therefore consider the following so as to enhance the rigour of this study.

Introduction

1. Line 60: insert “inadequate”

2. Line 90: dietary diversity is not a method.

3. Line 105 to 106: “Moreover, several studies have revealed that DD reduces mortality”. This sentence may not be accurate. It should rather be that “….increases in DD may reduce the risks of mortality.”

4. No mention has been made of the following article that was conducted in Bangladesh on the same sub-population. [https://doi.org/10.1093/cdn/nzab033_039].

Methods

1. Outcome variable

a) FAO guidelines have been referenced differently on lines 144 as citation (20) and 148 as citation (23).

b) Citation (23) does not cover FAO guidelines but rather “Appropriate body-mass index for Asian populations and its implications for policy and intervention strategies.”

c) The FAO guidelines were validated to measure diet quality for women of Reproductive age (15 to 49 years) and not for the age group in this study. The reviewer acknowledges that this has been stated as a limitation in the discussion section.

d) Although authors cited (21) and (22) as bases for using the Women Dietary Diversity Score (W-DDS) for men and women older than 60 years. Citation (21) is a publication on “Prevalence and socio-economic determinants of inadequate dietary diversity among adolescent girls and boys in Bangladesh.” While publication (22) is about extending W-DDS to children and adolescents aged 4-15 years…

2. Explanatory variable

a) How did the authors arrive at the inclusion of this limited number of variables as covariates? This is relevant because factors like oral health (i.e. presence of a problem with the gums or teeth), presence of a chronic illness such as diabetes or hypertension, cognitive status, belonging to a social support group, among other factors, have been shown to affect the nutritional status of the elderly in a myriad of publications. But conspicuously, none of these are included in this study.

b) Provide a brief explanation of how food security was assessed.

c) Also, explain how wealth was assessed, briefly.

Results

1. Table 2

a) The headings for column 2, 3, and 4 may need to be edited. Because the columns also contain mean and SD.

b) For rows 3 and 4, the differences in mean between men and women could also be determined and the p-value provided.

2. Tables 3 and 4

a) The assessment and inclusion of both income, categorised as no income, <8000 or ≥8000 and wealth grouped as quintiles may exert multicollinearity effects on your regression models.

b) Both cash income and wealth (I assume assessed using household assets) usually measure the same concept. Consider re-running your multivariable analyses. Or authors should provide the dataset that supports these findings for the reviewer to ascertain the presence or absence of multicollinearity.

**Reviewer**	**2**
**Name**	**Thomas, Roger**
**Affiliation**	**University of Calgary, Family Medicine**
**Date**	**01-Dec-2025**
**COI**	**None**

Methods

You wrote: “10 food groups (grains, white roots, tubers, and plantains; 142 pulses: beans, peas, and lentils; nuts and seeds; dairy; meat, poultry, and fish; eggs; dark green 143 leafy vegetables; other vitamin A-rich fruits and vegetables; other vegetables; and other fruits), 144 following the FAO guidelines for women (20). Diet quality and diversity were estimated by 145 the consumption of foods from various food groups, the prevalence of inadequate DD (defined 146 as consumption of less than five food groups out of 10), and the DD score (consumption of the 147 actual number of food groups) using a 24-hour recall period according to the FAO guidelines 148 (23), but used for other age and sex groups too (21,22).”

[How did you know if the respondents understood what terms such as “white roots, tubers and plantains” mean? Did you show them the vegetables?

[You used 24 hour recall. This would be interpreted by the subjects as only what they ate in the past 24 hours. So how did you assess the frequency and quantity of each food group consumed for e.g. a month. If they only ate it once a month as a treat it would be very different form daily consumption]

[How available is each food group during the year? I worked in Malawi and 400 miles from the coast but we were still influenced by the monsoons and what was on sale in the markets by individuals offering e.g. just a few small tomatoes varied a lot by the month]

Results

[Table 2 shows statistical differences by gender due to the large sample but many of the differences are only 1, 2 or 3 % points. Comment in detail and emphasise these clinically not significant findings].

Once you break the results down in Tables 3 and 4 by food insecurity, household wealth and age not surprisingly the differences are striking and should be emphasised.

Discussion

Good discussion of effects of urbanisation on food opportunities and preferences and male/female composition of households.

VERSION 1 - AUTHOR RESPONSE

**Comments**	**Responses**
**Reviewer: 1 [Mr. Hammond Addae Yaw, Nursing & Midwifery Training Colege, Kpembe, Kwame Nkrumah University of Science and Technology]**	
**Summary**<br>Thank you for the opportunity to review this manuscript.<br>This is an important study that was conducted among a population that is often overlooked, yet are becoming a sub-group that requires nutritional attention. The sample size is a strength; however, methodological approaches may limit the thoroughness of this study. Specifically, the non-inclusion of equally important and well-known nutrition-related geriatric factors, such as cognitive status, or the presence or otherwise of chronic diseases (e.g. hypertension, diabetes etc.), is a main flaw. The authors may therefore consider the following so as to enhance the rigour of this study.	Thank you for highlighting this point. We have acknowledged the absence of cognitive status, chronic disease indicators, and other geriatric nutrition-related variables as limitations in the 'Strengths and limitations of this study' section and the Discussion. Please see last paragraph of Discussion section.
**Introduction**	
1. Line 60: insert "inadequate"	Thank you. We already excluded this entire section based on the editorial comment.
2. Line 90: dietary diversity is not a method.	Thank you for pointing this out. We revised the Introduction section accordingly. Please see the first paragraph of Introduction section.
3. Line 105 to 106: "Moreover, several studies have revealed that DD reduces mortality". This sentence may not be accurate. It should rather be that "….increases in DD may reduce the risks of mortality."	Thank you. We revised the sentence to reflect that increased dietary diversity is associated with reduced mortality risk. Please see the second paragraph of Introduction section.
4. No mention has been made of the following article that was conducted in Bangladesh on the same sub-population. [https://doi.org/10.1093/cdn/nzab033_039].	Thank you for spotting this. The cited work was a conference abstract based on the same dataset that we used for this manuscript. The present manuscript developed the work with a focus on dietary diversity as the outcome variable and expanded it into a full manuscript, which we then submitted to this journal.
**Methods**	
1. Outcome variable	a) Thank you so much for noticing this. We corrected the references in the revised version.

Comments	Responses
a) FAO guidelines have been referenced differently on lines 144 as citation (20) and 148 as citation (23).<br><br>b) Citation (23) does not cover FAO guidelines but rather "Appropriate body-mass index for Asian populations and its implications for policy and intervention strategies."<br>c) The FAO guidelines were validated to measure diet quality for women of Reproductive age (15 to 49 years) and not for the age group in this study. The reviewer acknowledges that this has been stated as a limitation in the discussion section.<br>d) Although authors cited (21) and (22) as bases for using the Women Dietary Diversity Score (W-DDS) for men and women older than 60 years. Citation (21) is a publication on "Prevalence and socio-economic determinants of inadequate dietary diversity among adolescent girls and boys in Bangladesh." While publication (22) is about extending W-DDS to children and adolescents aged 4-15 years…	b) Thank you. We revised the list of references in the updated manuscript.<br><br>c) Thank you. We acknowledge that MDD-W was originally validated for women of reproductive age and have noted this as a limitation.<br><br>d) Thank you for this observation. We clarified this point in the Methods and highlighted the lack of validation for older adults in the limitations.
2. Explanatory variable<br>a) How did the authors arrive at the inclusion of this limited number of variables as covariates? This is relevant because factors like oral health (i.e. presence of a problem with the gums or teeth), presence of a chronic illness such as diabetes or hypertension, cognitive status, belonging to a social support group, among other factors, have been shown to affect the nutritional status of the elderly in a myriad of publications. But conspicuously, none of these are included in this study.<br>b) Provide a brief explanation of how food security was assessed.<br><br>c) Also, explain how wealth was assessed, briefly.	a) Thank you for raising this point. These variables were not collected in the original national survey and therefore could not be included in the analysis. We have acknowledged their absence as a limitation in the Strengths and limitation section and highlighted them as areas for future research.<br><br>b) Thank you. We added an explanation of how food security was assessed using the Household Food Insecurity Access Scale (HFIAS) tool (Reference 24) in the Explanatory Variables subsection.<br><br>Ref 24: Coates J, Swindale A, Bilinsky P. Household Food Insecurity Access Scale (HFIAS) for Measurement of Food Access: Indicator Guide: Version 3: (576842013-001) [Internet]. 2007 [cited 2026 Jan 4]. Available from: https://doi.apa.org/doi/10.1037/e576842013-001<br><br>c) Thank you. We added a brief description in the manuscript noting that wealth was assessed using MEASURE DHS (Monitoring and Evaluation to Assess and Use Results Demographic and Health Surveys) for Demographic Health Surveys were

Comments	Responses
	followed (Reference 25) (see Explanatory variables subsection).<br><br>Ref 25: National Institute of Population Research and Training (NIPORT), Mitra and Associates, and ICF International. Bangladesh Demographic and Health Survey 2014. Dhaka, Bangladesh, and Rockville, Maryland, USA: NIPORT, Mitra and Associates, and ICF International; 2016 p. 354.
**Results**	
1. Table 2<br>a) The headings for columns 2, 3, and 4 may need to be edited. Because the columns also contain mean and SD.<br>b) For rows 3 and 4, the differences in mean between men and women could also be determined and the p-value provided.	a) Thank you. We addressed the issue. Please see Table 2.<br><br>b) Thank you. The p-value for mean differences in mean between men and women has been added. Please see Table 2.
2. Tables 3 and 4<br>a) The assessment and inclusion of both income, categorised as no income, <8000 or ≥8000 and wealth grouped as quintiles may exert multicollinearity effects on your regression models.<br>b) Both cash income and wealth (I assume assessed using household assets) usually measure the same concept. Consider re-running your multivariable analyses. Or authors should provide the dataset that supports these findings for the reviewer to ascertain the presence or absence of multicollinearity.	a) We added description of multicollinearity analysis. As per multicollinearity analysis income and wealth quintiles show very weak correlation.<br><br>b) Thank you for raising this important point. Although cash income and household wealth can capture related aspects of socioeconomic status, they represent different constructs. Individual income reflects short-term earnings of the respondent, whereas household wealth reflects longer-term asset accumulation and living conditions. To address the concern about potential multicollinearity between these variables, we examined the correlation matrix for all explanatory variables. The correlation between income category and wealth quintiles was very low (correlation co-efficient: 0.0416), indicating that these variables were not strongly correlated in our dataset. Since no strong correlations were identified (most of the correlation co-efficient were <0.1 and only few were between 0.1 and 0.4), we did not exclude any variables on the basis of multicollinearity, and both income and wealth were retained in the multivariable models. Please see the Statistical analysis subsection. As requested, the dataset is provided in Supplementary File 2.
**Reviewer: 2 [Prof. Roger Thomas, University of Calgary]**	
**Methods**	
You wrote: "10 food groups (grains, white roots, tubers, and plantains; 142 pulses: beans, peas, and lentils; nuts and seeds; dairy; meat, poultry,	

Comments	Responses
and fish; eggs; dark green 143 leafy vegetables; other vitamin A-rich fruits and vegetables; other vegetables; and other fruits), 144 following the FAO guidelines for women (20). Diet quality and diversity were estimated by 145 the consumption of foods from various food groups, the prevalence of inadequate DD (defined 146 as consumption of less than five food groups out of 10), and the DD score (consumption of the 147 actual number of food groups) using a 24-hour recall period according to the FAO guidelines 148 (23), but used for other age and sex groups too (21,22)."<br><br>a) [How did you know if the respondents understood what terms such as "white roots, tubers and plantains" mean? Did you show them the vegetables?<br><br>b) [You used 24 hour recall. This would be interpreted by the subjects as only what they ate in the past 24 hours. So how did you assess the frequency and quantity of each food group consumed for e.g. a month. If they only ate it once a month as a treat it would be very different from daily consumption]<br><br>c) [How available is each food group during the year? I worked in Malawi and 400 miles from the coast but we were still influenced by the monsoons and what was on sale in the markets by individuals offering e.g. just a few small tomatoes varied a lot by the month]	a) Thank you. Respondents were not asked about food group names. They were asked to mention the food items they consumed in the previous 24 hours. Trained data collectors then assigned each item to the appropriate food group based on the MDD-W guideline.<br><br>b) Thank you for your comment. We used a 24-hour recall method to record whether participants consumed any items from each food group at least once in the previous day, following the MDD-W guideline. Respondents reported the specific food items they ate, and we coded each item into the appropriate FAO food group. As per the MDD-W guideline, we did not collect information on the frequency or quantity of foods consumed, our measure captures only whether a food group was consumed at least once in the 24-hour period.<br><br>c) Thank you for raising this important point. We acknowledge that seasonal variation can affect the availability of certain foods. However, in this survey, data were collected across all seasons and from all regions (divisions) of Bangladesh. Because data collection covered the full calendar year and diverse settings, we expect that seasonal fluctuations in the availability of foods such as fruits, and vegetables were minimized in our final dietary diversity estimates.
**Results**	
[Table 2 shows statistical differences by gender due to the large sample but many of the differences are only 1, 2 or 3 % points. Comment in detail and emphasise these clinically not significant findings].	Thank you for your comment. As shown in Table 2, only three indicators differed significantly between men and women: adequate dietary diversity (4.93 percentage points), fish consumption (6.8 percentage points), and egg consumption (4.97 percentage points). For each of these, the absolute differences were more than 4 percentage points. For all other food groups, the differences between men and women were small and were not statistically significant. We clarified this distinction in the Results section and noted in the Discussion that small numerical differences should be interpreted cautiously. Please see the Discussion section.

Comments	Responses
Once you break the results down in Tables 3 and 4 by food insecurity, household wealth and age not surprisingly the differences are striking and should be emphasised.	Thank you for this comment. We have emphasised the associations of food insecurity, wealth, and age with inadequate dietary diversity in both the Results and Discussion sections. In the Results section, we highlighted how these factors showed stronger and more distinct patterns once sex-stratified analyses were performed. For example, older age and marital status were associated factors among men, whereas middle-wealth status and younger age were associated factors among women. Similarly, different levels of household food insecurity were associated with inadequate DD in both sexes but with varying strengths of association. In the Discussion section, we further interpreted these sex-specific patterns and explained their potential implications for dietary behaviour among older adults in Bangladesh.
**Discussion**	
Good discussion of effects of urbanisation on food opportunities and preferences and male/female composition of households.	Thank you for your comment.

VERSION 2 - REVIEW

**Reviewer**	**2**
**Name**	**Thomas, Roger**
**Affiliation**	**University of Calgary, Family Medicine**
**Date**	**11-Jan-2026**
**COI**	

I reviewed this manuscript on 1 December 2025 and the authors have made appropriate small changes (in yellow) in response to my comments
